# Proton beam therapy for external auditory canal and middle ear cancers

**DOI:** 10.1093/jrr/rrag024

**Published:** 2026-04-22

**Authors:** Masanori Machida, Takashi Ono, Koki Ando, Rei Nishikawa, Yuntao Dai, Takuya Tominaga, Yusuke Azami, Yoshiaki Takagawa, Motohisa Suzuki, Ichiro Seto, Kanako Takayama, Tatsuhiko Nakasato, Yasuhiro Kikuchi, Masao Murakami

**Affiliations:** Department of Radiation Oncology, Southern TOHOKU Proton Therapy Center, 7-172 Yatsuyamada, Koriyama, Fukushima, 963-8052, Japan; Department of Radiology, Division of Radiation Oncology, Faculty of Medicine, Yamagata University, 2-2-2 Iida-Nishi, Yamagata City, Yamagata 990-9585, Japan; Department of Radiation Oncology, Southern TOHOKU Proton Therapy Center, 7-172 Yatsuyamada, Koriyama, Fukushima, 963-8052, Japan; Department of Radiation Oncology, Southern TOHOKU Proton Therapy Center, 7-172 Yatsuyamada, Koriyama, Fukushima, 963-8052, Japan; Department of Radiation Oncology, Southern TOHOKU Proton Therapy Center, 7-172 Yatsuyamada, Koriyama, Fukushima, 963-8052, Japan; Department of Radiation Oncology, Southern TOHOKU Proton Therapy Center, 7-172 Yatsuyamada, Koriyama, Fukushima, 963-8052, Japan; Department of Radiation Oncology, Southern TOHOKU Proton Therapy Center, 7-172 Yatsuyamada, Koriyama, Fukushima, 963-8052, Japan; Department of Radiation Oncology, Southern TOHOKU Proton Therapy Center, 7-172 Yatsuyamada, Koriyama, Fukushima, 963-8052, Japan; Department of Radiation Oncology, Southern TOHOKU Proton Therapy Center, 7-172 Yatsuyamada, Koriyama, Fukushima, 963-8052, Japan; Department of Radiation Oncology, Southern TOHOKU Proton Therapy Center, 7-172 Yatsuyamada, Koriyama, Fukushima, 963-8052, Japan; Department of Radiation Oncology, Southern TOHOKU Proton Therapy Center, 7-172 Yatsuyamada, Koriyama, Fukushima, 963-8052, Japan; Department of Radiation Oncology, Southern TOHOKU Proton Therapy Center, 7-172 Yatsuyamada, Koriyama, Fukushima, 963-8052, Japan; Department of Radiation Oncology, Southern TOHOKU Proton Therapy Center, 7-172 Yatsuyamada, Koriyama, Fukushima, 963-8052, Japan; Department of Radiation Oncology, Southern TOHOKU Proton Therapy Center, 7-172 Yatsuyamada, Koriyama, Fukushima, 963-8052, Japan

**Keywords:** proton beam therapy, external auditory canal cancer, middle ear cancer

## Abstract

Few studies have evaluated the efficacy of proton beam therapy (PBT) for external auditory canal cancer (EACC) or middle ear cancer (MEC). Therefore, the present study aimed to evaluate the efficacy and toxicity of PBT for EACC and MEC. Between December 2009 and August 2018, 15 patients (seven males and eight females) underwent PBT for EACC or MEC. The median patient age was 64 years (range: 40–82 years). Ten patients had EACC, and five patients had MEC. PBT was administered using the passive scattering method. The median total dose of the BED10 was 88.8 Gy relative biological effectiveness (RBE) (range = 85.6–99.3 Gy [RBE]), administered in 2 Gy fractions (EQD2) (α/β = 10). Chemotherapy was conducted in 14 patients, systemic chemotherapy in five and intra-arterial infusion chemotherapy in nine. The median follow-up period for all patients was 60 months (range: 7–136 months). The 3-year overall survival, local control and progression free survival rates were 65%, 66.7% and 53.3%, respectively. Four patients had grade 3 hearing impairment owing to late toxicity. However, these patients had hearing impairment before treatment as well. No grade 4 or higher late toxicity was observed during the follow-up period. PBT is an effective treatment for EACC and MEC with tolerable toxicity, including in the setting of combined chemotherapy.

## INTRODUCTION

External canal ear cancer (EACC) and middle ear cancer (MEC) are rare neoplasms. The frequency of these diseases is estimated to be 1–6 per million population [1]. EACC accounts for ˂0.2% of all head and neck cancers [2]. Given this rarity, many previous studies have had small sample sizes. Although the importance of treatment at an early stage has been emphasized, the symptoms are nonspecific and tend to be confused with chronic otitis [3]. Consequently, diagnosis is delayed in many cases, and treatment is often initiated at an advanced stage. Due to the rarity of this disease, no standard treatment has yet been established. Generally, surgical procedures, including lateral temporal bone resection or radiotherapy alone, are indicated for early-stage cancer. Advanced-stage cancer is indicated for surgical procedures and postoperative radiotherapy, including subtotal and total bone resections [4]. However, despite their invasiveness, these procedures do not lead to good outcomes in advanced-stage cancer. Owing to the anatomical complexity and proximity of the temporal bone to vital organs, fatal perioperative complications commonly occur. In addition, postoperative functional and cosmetic problems should not be ignored.

In addition to systemic chemotherapy, intra-arterial infusion chemotherapy (IAIC) is indicated for advanced head and neck cancer. Regarding the treatment of EACC, prior reports have described concomitant chemoradiotherapy (CCRT) using systemic chemotherapy and IAIC [5, 6]. In addition, catheterization through a superficial temporal artery (STA) or occipital artery (OA) was developed [7]. Mitsudo *et al.* reported not only good treatment results, but also functional and aesthetic preservation in oral carcinomas using IAIC via STA [8]. Our institution has similarly reported treatment outcomes using IAIC and proton beam therapy (PBT) for head and neck cancers [9–11]. These reports suggest that CCRT has the potential to improve the outcomes of head and neck cancer.

Particle-beam radiotherapy displays a better dose distribution because of the rapid dose fall-off at the distal end, known as the Bragg peak [12]. Because this property reduces toxicity to normal organs and provides high-dose irradiation to the tumour volume, it may be an appropriate treatment for head and neck cancers. In recent years, carbon-ion radiotherapy (CIRT) has been reported to lead to good outcomes in EACC and MEC, and multicentre and retrospective case series studies of CIRT have been published [13, 14]. However, there are relatively few reports on PBT for EACC and MEC. Herein, we aimed to evaluate the outcomes of PBT in patients with EACC and MEC.

## MATERIAL AND METHODS

### Patients and study design

We collected clinical data from the electronic medical records of patients at our institution. Fifteen patients who underwent PBT for EACC and MEC between December 2009 and August 2018 were enrolled in this study. The inclusion criteria were as follows: malignant pathology, regardless of histological type; a medical history of PBT for EACC and MEC with no concomitant use of photon therapy; no medical history of radiotherapy (RT) for a head and neck cancer: no distant metastasis (this criteria did not apply to patients with the lung metastasis of adenoid cystic carcinoma [ACC], which have good prognosis [15]). The patients were also required to have an Eastern Cooperative Oncology Group performance status (ECOG-PS) of 0–2, and to have adequate haematological parameters (white blood cell count >2000/μL: haemoglobin >9 g/dL; platelet count >1 × 10^6^/μL). All patients underwent physical examination, magnetic resonance imaging (MRI) and positron emission tomography-computed tomography (PET-CT) for tumour staging. All primary tumours were classified according to the Union for International Cancer Control TNM classification of malignant Tumours, 8^Th^ edition [16]. The acute and late toxicities were evaluated by Common Terminology Criteria for Adverse Events version 5.0 (CTCAE ver.5.0). Excel statistical analysis software (Microsoft, USA) was used for all statistical analyses. This study was conducted in accordance with the principles of the Declaration of Helsinki and was approved by the ethics committee of our institution (approval number: 289).

### Proton beam therapy

Treatment planning was based on a CT planning system (Xio-M; MCS Japan, Tokyo, Japan). The gross tumour volume (GTV) was determined using computed tomography (CT), MRI and PET-CT. The clinical target volume (CTV) was defined as the GTV plus a margin of 3–5 mm. The CTV was finalized by considering the extent of the clinical tumour extension. The planning target volume (PTV) was defined as CTV plus a margin of 3 mm. Prophylactic irradiation of the cervical lymph node region on the affected side was conducted in patients with lymph node metastasis. In addition, it was performed even in cN0 cases when the radiation oncologist judged it to be necessary. The proton beam was adjusted to the appropriate angle to avoid normal tissue and to ensure a high dose of radiation to the tumour using the dual-portal broad beam method, and multi-leaf collimators were used five times a week. We used two or three proton beam portals with energy levels of 150 MeV and 210 MeV. We prescribed a dose at the isocentre that covered 90% of the PTV. We defined the PBT dose as the physical dose multiplied by the relative biological effectiveness (RBE) value of 1.1, and described it in units of Gy (RBE). As various doses were prescribed for treatment, the biological effects of PBT were assessed using BED. BED was calculated with the linear-quadratic model to compare the treatment effects and late complications amongst different dose and fractionation schedules. BED Gy (RBE) = total dose × (1 + dose per fraction/(α/β)). α/β ratios of 10 Gy (RBE) and 3 Gy (RBE) were applied to tumours and normal tissues, respectively. EQD(α/β)/2 signifies the equivalent dose as 2-Gy fractions for a given α/β value. EQD(α/β)/2 was calculated according to the following equation: EQD(α/β)/2 = BED/ (1 + 2/(α/β)).

Based on these calculations, the normal tissue dose constraints were defined and evaluated using EQD2 (α/β = 3) values as follows: Brainstem maximum dose <60Gy (RBE); Spinal cord maximum dose <45 Gy (RBE); Temporal lobe maximum dose <70 Gy (RBE); Mandible maximum dose <70 Gy (RBE); Optic nerve maximum dose <54 Gy (RBE); Optic chiasm maximum dose <70 Gy (RBE). Depending on the judgement of the treating radiation oncologist, the priority was given to tumour coverage rather than to strict adherence to normal tissue dose constraints.

### Intra-arterial infusion chemotherapy

We used two methods of IAIC: catheterization through the STA or OA, and a transfemoral approach using the Seldinger method. Before treatment, 3-dimensional CT angiography of the carotid artery was conducted to determine the feeding artery based on tumour extension. In general, the feeding artery is the posterior auricular artery and/or STA. In addition, depending on the extent of tumour and lymph node metastasis, other vessels could be feeding arteries, such as the transverse facial artery, OA and maxillary artery. When using the STA or OA approaches, the catheter tip is placed in the external carotid artery. Digital subtraction angiography (DSA) and flow-check MRI were conducted to ensure that the entire tumour was enhanced. This suggests that the anti-cancer drug, cisplatin, covers the entire tumour. Cisplatin was administered in doses of 50–70 mg/body over 5 h, once a week, three to seven times. During IAIC, sodium thiosulfate, which neutralizes cisplatin toxicity, was administered intravenously at 8 g/m^2^ over 8 h, and the dose was adjusted according to the dose of cisplatin. One patient received docetaxel in addition to cisplatin to increase the treatment effectiveness. When the Seldinger method was used via the femoral artery, the feeding arteries were selected using a microcatheter. Following the selection of each blood vessel, DSA and cone beam CT (CBCT) were conducted to confirm enhancement of the entire tumour. The dose of cisplatin was 80–100 mg/m^2^, which was determined based on the patient’s renal function. Sodium thiosulfate was simultaneously administered during cisplatin infusion into each feeding artery. The procedure was conducted 3–4 times, biweekly. After cisplatin administration, steroids were administered via catheter to prevent toxicity.

Five patients received systemic chemotherapy, and three received oral fluoropyrimidine (TS-1) at a daily dose of 50–60 mg/m^2^. One patient received 2 cycles of 5-fluorouracil (800 mg/m^2^) and nedaplatin (130 mg/m^2^), followed by three cycles of cisplatin and docetaxel. One patient was treated with two cycles of docetaxel (60 mg/m^2^), nedaplatin (700 mg/m^2^) and 5-Fluorouracil (100 mg/m^2^). The second cycle of chemotherapy was administered at a 25% dose reduction due to grade 4 neutropenia.

### Statistical analysis

Local control (LC), overall survival (OS) and progression free survival (PFS) rates were calculated. LC was defined as the period with no local recurrence at the primary site from the start of PBT. OS was defined as the period between the start of PBT and the date of death, or the last confirmed date of survival. PFS was defined as the period without any increase in the size of the tumour lesions or the development of new lesions. The LC, OS and PFS rates were calculated using the Kaplan–Meier method, and were compared amongst the subgroups by univariate analysis using the log-rank test. Statistical significance was set at *P* < 0.05. The following factors were evaluated for their potential involvement with OS, LC and PFS: age (≧64 years vs.<64 years), sex (male vs. female), primary site (external auditory canal vs. middle ear), lymph node status (positive vs. negative), total radiation dose (≧ BED10 88.8Gy[RBE] vs. < BED10 88.8Gy[RBE]), histological type (squamous cell carcinoma vs. other type), and chemotherapy (IAIC vs. others), respectively. Late toxicity was defined as the appearance of these symptoms 3 months after the end of PBT. Acute toxicity was defined as the early appearance of symptoms.

## RESULTS

Patient characteristics are summarized in Table 1. One patient had T2 disease, three had T3 disease, 10 had T4a and 1 had T4b disease. One patient had recurrent lesions after surgery at another hospital and was classified as rT3N0M0. Four patients were diagnosed with lymph node metastasis. The median total dose of the BED10 was 88.8 Gy (RBE) (range = 85.6–99.3 Gy [RBE]). The characteristics of the treatments are summarized in Table 2. Overall, 14 patients received concurrent chemotherapy, five underwent systemic chemotherapy, seven patients underwent IAIC via the STA or OA, and two patients underwent IAIC through the femoral artery using the Seldinger method.

**Table 1:** Characteristics

Characteristics (*n* = 15)	
Number of patients	15
Median age (range)	64 (40–82)
Gender	
Male/Female	7/8
PS (ECOG)	
0/1/2	1/12/2
Primary site	
External auditory canal/ Middle ear	10/5
Tumour type	
Squamous cell carcinoma	9
Adenoid cystic carcinoma	3
The others	3
Stage (UICC 8th)	
II/ III/ IVa/ IVb	1/3/10/1
T stage	
T2/ T3/ T4a/ T4b	1/5/2/7
N stage	
N0/ N1/ N2b	11/1/3
M stage	
M0/ M1	14/1

Abbreviations: PS, Perfomance status; ECOG, Eastern Cooperative Oncology Group; UICC, Union for International Cancer Control.

**Table 2:** Treatment

Treatment	Number of patients or Median (Range)
Prescribed dose/ Fractionation	
70.4 GyE(RBE)/ 32 Fr	3
72.6 GyE(RBE)/ 33 Fr	2
74 GyE(RBE)/ 37 Fr	2
70.2 GyE(RBE)/ 32 Fr	1
74.8 GyE(RBE)/ 34 Fr	3
77 Gy(RBE)/ 35 Fr	1
76.8 Gy(RBE)/ 32 Fr	1
44 Gy(RBE)/20 Fr + 28 Gy(RBE)/14 Fr	1
81.4 Gy(RBE)/ 37 Fr	1
Total radiation dose	
BED10	88.8 (85.6–99.3) Gy(RBE)
Types of chemotherapy	
None	1
Systemic chemotherapy	5
Intra-arterial infusion chemotherapy	9

Abbreviations: BED, biological effective dose; EQD2, Equivalent Dose in 2Gy fraction; RBE, Relative biological effectiveness.

**Fig. 1:**
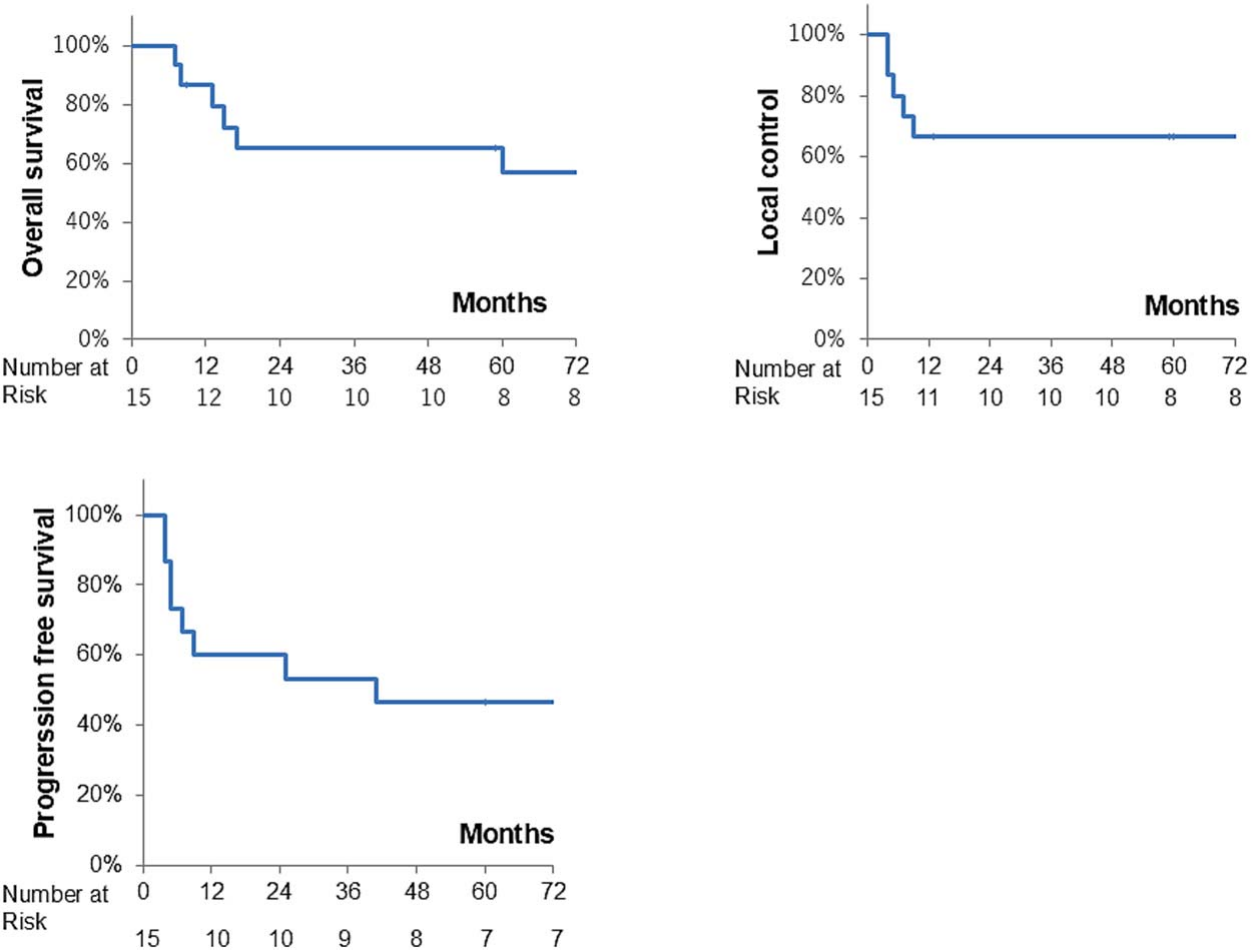
Kaplan–Meier curves of overall survival rate, local control rate and progression free survival rate of all patients (*n* = 15).

**Fig. 2:**
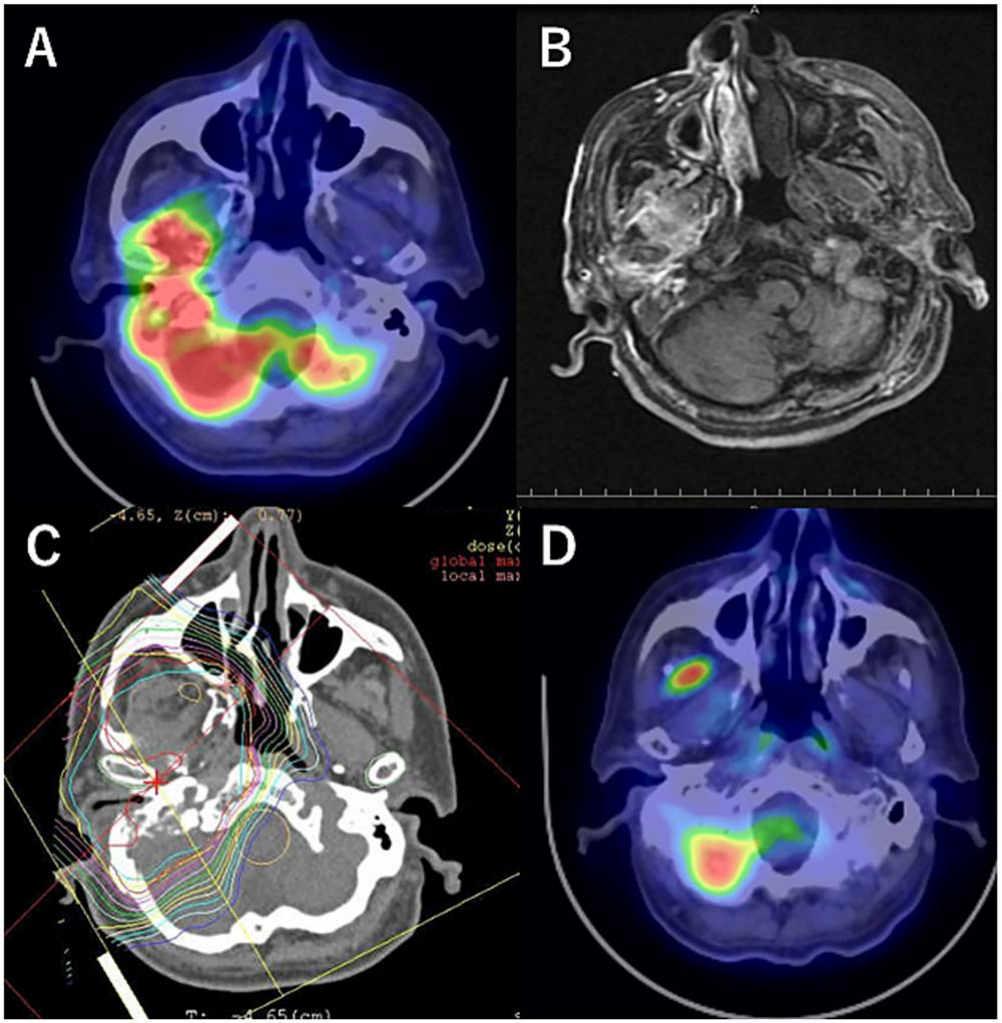
Representative case of a T4bN0M0 MEC treated with proton beam therapy (PBT) and intra-arterial infusion chemotherapy via superficial temporal artery (STA). (A) Image of fluorodeoxyglucose-positron emission tomography (18F-FDG-PET/CT) before PBT. (B) Flow check magnetic resonance imaging showed the entire tumour was enchanced. (C) Dose distribution of PBT planning. (D) PET/CT performed 1 year after PBT showed that the FDG uptake of tumour was disappeared. The FDG uptake in masticator space reflected inflammation.

The median observation period was 60 months (range:7–136 months). The 3-year OS, PFS and LC rates were 65% (95% CI: 40.1–89.6), 53.3% (95% CI: 28.1–78.6), 66.7% (95 CI: 42.8–90.5), respectively. The 5-year OS, PFS and LC rates were 55.7% (95% CI: 28.6–82.9), 46.7% (95% CI: 21.4–71.9), 66.7% (95 CI: 42.8–90.5), respectively (Fig. 1). Representative cases of MEC treated with PBT and IAIC via the STA are presented in Fig. 2. Six patients died during the follow-up period. One patient, whose cancer was fully controlled, died of pneumonia; four patients died of local recurrence after treatment; and one patient died of lung metastasis (which was present before treatment initiation). However, the primary site of the PBT treatment was controlled. Detailed information for each case, including histological subtype, primary site, stage, the irradiated field and dose, chemotherapy regimens and sites of recurrence, is summarized in Table 3.

**Table 3:** Detailed information for each case

Case	Age	Sex	Histological type	Primary site	TMN	Stage	Irradiation field	Chemotherapy	Recurrence site
1	40	F	ACC	EACC	rT3N0M0	III	76.8Gy(RBE)/32Fr to primary site	none	NED
2	70	F	ACC	EACC	T3N0M0	III	70.4Gy(RBE)/32Fr to primary site	Intra-arterial CDDP through STA	NED
3	56	F	SCC	EACC	T3N0M0	III	74.8Gy(RBE)/34Fr to primary site	TS-1	NED
4	82	M	SCC	MEC	T4bN0M0	IVa	81.4Gy(RBE)/37Fr to primary site	Intra-arterial CDDP through STA	NED
5	51	M	SCC	EACC	T2N0M0	II	72.6Gy(RBE)/33Fr to primary site	Intra-arterial CDDP through STA	NED
6	69	M	SCC	EACC	T3N2bM0	IVa	44Gy(RBE)/20Fr to the prophylactic field, 30.8Gy(RBE)/14Fr to primary site for the boost	Intra-arterial CDDP and DTX through STA	NED
7	49	F	SCC	EACC	T4aN2bM0	IVa	40Gy(RBE)/20Fr to the prophylactic field, 30Gy(RBE)/15Fr to primary site,34Gy(RBE)/17Fr to metastatic lymph nodes for the boost	Intra-arterial CDDP through STA	NED
8	67	M	Adenocarcinoma	EACC	T4bN0M0	IVa	70.2Gy(RBE)/32Fr to primary site	Intra-arterial CDDP through STA	lung metastases
9	64	F	SCC	EACC	T4aN0M0	IVa	74Gy(RBE)/37Fr to primary site	Intra-arterial CDDP through FA	local recurrence
10	67	M	Atypical carcinoid Tumour	EACC	T4bN0M0	IVa	70.4Gy(RBE)/32Fr to primary site	Intra-arterial CDDP through STA	Multiple distant metastases
11	66	F	unknown	MEC	T4bN0M0	IVa	74.8Gy(RBE)/34Fr to primary site	TS-1	local recurrence
12	75	M	SCC	MEC	T4bN1M0	IVa	48.4Gy(RBE)/22Fr to the prophylactic field, 22Gy(RBE)/10Fr to primary site and metastatic lymph nodes for the boost	TS-1	local recurrence
13	44	M	SCC	MEC	T4bN0M0	IVa	44Gy(RBE)/20Fr to the prophylactic field, 28.6Gy(RBE)/13Fr to primary site and metastatic lymph nodes for the boost	5-FU and nedaplatin, followed by cisplatin and docetaxel	local recurrence
14	61	F	ACC	EACC	T3N2bM1	IVb	48.4Gy(RBE)/22Fr to the prophylactic field, 28.6Gy(RBE)/13Fr to primary site and metastatic lymph nodes for the boost	Intra-arterial CDDP through STA	Progression of lung metastases
15	51	F	SCC	MEC	T4bN0M0	IVa	44Gy(RBE)/20Fr to the prophylactic field, 28Gy(RBE)/14Fr to primary site and metastatic lymph nodes for the boost	docetaxel, nedaplatin, and 5-FU	leptomeningeal metastasis

Abbreviations: F, female; M, male; SCC, Squamous cell carcinoma; ACC, adenoid cystic carcinoma; EACC, external ear cancer; MEC, middle ear cancer; TS-1, superfical temporal artery; TS-1, oral fluoropyrimidine; CDDP, cisplatin; DTX, docetaxel; 5-FU, 5-fluorouracil; NED, no evidence disease.

Amongst the factors evaluated for their potential involvement in OS, LC and PFS, the primary site and type of chemotherapy were statistically significant (Table 4). The acute and late toxicities observed during the treatment and follow-up periods are shown in Table 5. Regarding the acute toxicity, grade 3 radiation dermatitis was observed in four patients, grade 3 hearing impairment was observed in one patient, grade 3 nausea was recorded in one patient, and grade 3 white blood cell count decrease and grade 4 neutropenia was observed in one patient who received systemic chemotherapy. No grade 5 toxic events were observed. Two patients discontinued the treatment because of grade 3 acute dermatitis. However, treatment was restarted after 5 and 14 days. Thirteen patients completed the treatment without interruption.

**Table 4:** Univariate analysis using the log-rank test

Factors	No. of patients	OS	LC	PFS
		*P-value*	*P-value*	*P-value*
Age		0.8837	0.7542	0.6418
≥64 years	8			
<64 years	7			
Sex		0.9614	0.7542	0.9760
male	7			
female	8			
Total radiation dose		0.7048	0.4138	0.9163
≥BED10 88.8Gy(RBE)	8			
< BED10 88.8Gy(RBE)	7			
Primary site		< 0.001^*^	0.0025^*^	0.0395^*^
external auditory canal	10			
middle ear	5			
Histological type		0.5886	0.3057	0.6184
SCC	9			
others	6			
Chemotherapy type		0.0841	0.0171^*^	0.2037
IAIC	9			
others	6			
Lymph node status		0.5347	0.7692	0.8992
positive	4			
negative	11			

Abbreviations: BED, biological effective dose; SCC, Squamous cell carcinoma; IAIC, intra-arterial infusion chemotherapy.

^^*^^ *P* < 0.05. Bold values indicate P < 0.05.

**Table 5:** The acute and late toxicity

	Grade		
	2	3	4
Acute		n (%)	
Radiation dermatitis	11 (73%)	4 (27%)	0
Oral mucositis	2 (13%)	0	0
Ear pain	4 (27%)	0	0
Middle ear inflammation	7 (47%)	0	0
Hearing impairment	6 (40%)	1 (7%)	0
Trigeminal nerve disorder	2 (13%)	0	0
Nausea	1 (7%)	1 (7%)	0
Neutrophil count decreasing	0	0	1 (7%)
White blood cell decreasing	0	1 (7%)	0
Anaemia	1 (7%)	0	0
Late			
Hearing impairment	3 (20%)	4 (27%)	0
Middle ear inflammation	4 (27%)	0	0
Headache	2 (13%)	0	0
Ear pain	1 (7%)	0	0
Facial nerve disorder	1 (7%)	0	0
Glossopharyngeal nerve disorder	1 (7%)	0	0

Regarding late toxicities, grade 3 hearing impairment was observed in four patients. However, eight patients had pretreatment symptoms of hearing impairment. It was therefore difficult to determine whether the hearing loss was due to irradiation or tumour invasion. No grade 3 or higher toxicities, other than hearing impairment, were observed. External auditory canal stenosis and occlusion were observed in two patients. No neurological complications were observed in patients treated with IAIC. All the patients were able to preserve their auricles.

## DISCUSSION

To the best of our knowledge, this is the first study to evaluate the efficacy and toxicity of PBT for malignant tumours of the external auditory canal and middle ear. Table 6 presents the results of our and prior studies, showing that they were comparable in terms of treatment outcomes. Ogawa *et al.* previously reported that the 5-year actuarial OS rate was 55% and the DFS rate was 54% for patients with EACC and MEC in a multicentre study [17]. According to a meta-analysis of 752 patients with external auditory canal squamous cell carcinoma (SCC), the 5-year OS rate was 57%, whilst the 5-year OS rate of patients treated with definitive CRT was 43.6% [18]. The outcomes of conventional radiotherapy, hypofractionated stereotactic radiotherapy, CIRT to EACC and MEC were reported, respectively [14, 19, 20]. Although a large proportion of the cases included stage III–IV disease, we demonstrated that the outcomes of EACC and MEC treated with PBT were comparable to those reported previously (Table 5). However, the present study encompassed a diverse range of cases, including recurrent disease and different histological types. In particular, ACC, which is characterized by slow progression, accounted for 20% of the cases and may have contributed to prolonged OS. Most of the previously reported studies, except for that by Hayashi, have focused on the treatment of SCC; therefore, direct comparisons across studies should be made with caution.

**Table 6:** Comparison with previous reports

Author	Treatment	Number of cases	Median follow-up period (months)	Result		Late adverse events
Ogawa *et al.*	RT and Surgery +RT	87	42	T1 in RT group:5-year LC: 83%	5-year OS for all patient: 55%	G4 osteoradionecrosis one case
				T2 in RT group:5-year LC: 45%		G4 ulceration one case
				T3 in RT group: 5-year LC: 0%		
Pemberton *et al.*	RT	123	63.6	5-year LC:56%	5-year OS: 40%	Bone necrosis six cases
						Soft tissue necrosis two cases
Murai *et al.*	SRT	29	40	T1/2:3-year LC: 70%	T1/2:3-year OS: 56%	G3 Radiation-related dermatitis two cases
				T3:3-year LC: 50%	T3:3-year OS: 79%	G3 Soft-tissue necrosis one case
				T4:3-year LC: 20%	T4:3-year OS: 0%	
Koto *et al.*	CIRT	13	12	3-year LC: 54%	3-year OS: 40%	G3 temporal bone necrosis one case
						
Hayashi *et al.*	CIRT	31	18.4	3-year LC: 55%	3-year OS: 58.7%	G3 central nervous system necrosis one case
						
Present study	PBT	15	60	3-year LC: 66.7%	3-year OS: 65%	G3 hearing impairment four cases

Abbreviations: RT, radiationtherapy; SRT, stereotactic radiationtherapy; CIRT, carbon ion radiotherapy; PBT, proton beam radiotherapy; LC, local control; OS, overall survival.

Although surgery plays an important role in treating EACC and MEC, it is invasive. Perioperative complications included bleeding, infection, carotid artery thrombosis and cerebrospinal fluid leak [19, 21]. Previous reports have indicated a surgical mortality rate of 5–18.7% [19, 22]. Moreover, complete resection is difficult because of its anatomically complex nature and proximity to vital organs. In the report of Ouaz *et al.*, more than one-third of patients did not undergo complete resection [23]. Additionally, a positive surgical margin has previously been shown to be associated with a poor prognosis [24]. Despite the risks and difficulties associated with surgery, the prognosis of advanced EACC and MEC is not satisfactory. Moody *et al.* previously reported that the 2-year survival rates for squamous cell carcinoma of the temporal bone were as follows: T3 lesion, 50%; T4 lesion, 7%, respectively [2]. Pemberton *et al.* also demonstrated cancer-specific survival of patients with advanced stage was 44% and 44% at 5- and 10-years, respectively [19]. Many reports have shown poor outcomes in patients with T3 or T4 tumours. Compared with the high perioperative fatality rate, acute toxicities were acceptable and treatment-associated deaths were not observed in the present study. Due to its tolerability, all patients were able to complete the treatment. Regarding late toxicity, the rate of grade 3 facial nerve palsy was 0%, and there were no new occurrences of grade 3 hearing impairments. However, hearing loss or facial nerve palsy cannot be avoided in patients who undergo subtotal or total bone resection. This suggests that PBT has an advantage over surgical treatment in preserving the facial nerve and hearing ability.

Regarding cosmetic problems, only a few studies have examined auricular preservation. Trojanowski *et al.* reported the usefulness of an ALT free flap for the reconstruction of lateral skull base defects [25]. In their study, 17 patients with T3 and T4 lateral skull base malignancies were reviewed. In 3 of the 17 cases, the tumour originated in the auricle, and in 10 cases, in the peri-auricle skin. At least 13 patients required auricular resection. In our study, the auricles of all 15 patients were preserved. Aesthetically, PBT may be superior to the surgery alone.

The usefulness of PBT for head and neck cancer has been previously reported [26]; indeed, the characteristics of PBT may be advantageous for treating temporal bone malignancies. Pemberton *et al.* previously reported of the outcomes of conventional radiotherapy for EACC and MEC [19]. In their study, radiation-induced osteonecrosis and soft-tissue necrosis were observed in 5% and 2% of patients, respectively. Murai *et al.* reported grade 2 or grade 3 soft-tissue necrosis in 5 of 17 patients (29%) treated with hypofractionated stereotactic radiotherapy [20]. In the present study, no cases of grade 2 or higher radiation-induced osteonecrosis, soft tissue necrosis or radiation-induced late dermatitis were observed. Ogawa *et al.* revealed that one of 77 patients who received conventional RT developed grade 4 dermatitis [17]. The dose of PBT may contribute to a lower frequency of osteonecrosis, soft tissue necrosis and dermatitis.

In the present study, patients with MEC appeared to have lower OS and LC rates compared with those with EACC. The difference in the prognosis between the EACC and MEC remains controversial. Yin *et al.* revealed that the 5-year survival rate of extrinsic carcinoma (external to the isthmus) was higher than that of intrinsic carcinoma (isthmus to the middle ear) [27]. However, the number of cases in the advanced stage (stage III or IV) of intrinsic carcinoma was higher than that of extrinsic carcinoma. Yin *et al.* previously advocated that tumour location should not be the single factor influencing the survival rate. In the present study, all patients with MEC were at stage IVa and tended to be at an advanced stage [27]. Therefore, whether the tumour location is a prognostic factor remains controversial.

CRT plays an important role in the treatment of EACC. Takenaka *et al.* previously reported that the 5-year OS of patients who received preoperative CRT was 85.7%, compared to 53.5% for those who underwent surgery ± RT. They concluded that postoperative CRT might improve the survival of patients surgically treated for EACC [18]. Shinomiya *et al.* treated 10 patients with CCRT using a combination of cisplatin, 5-fluorouracil and docetaxel, reporting 5 year-OS rate and DFS rates of 60% each [5]. Thus, CRT is a significant option for EACC, especially in the advanced stages. In our study, most patients were treated with CCRT. The favourable outcomes may have been attributable not only to proton beam therapy but also to the combined use of chemotherapy.

There have been some reports of good outcomes with IAIC for head and neck cancers [28]. Fujiwara *et al.* reported that patients with locally advanced carcinomas of the EACC and MEC (T3 and T4) were successfully treated with IAIC [6]. The 2-year OS and PFS were 58.7% and 53.8%, respectively. An alternative approach involving catheter insertion into the target artery via the superficial temporal artery has also been reported [7]. We used both methods of intra-arterial chemotherapy based on the patient’s status. In our study, nine patients received IAIC. The patients treated with IAIC showed more favourable LC compared with those treated with other approaches. The IAIC has also been reported to be a good indication when the tumour does not extend to the opposite side over the midline [29]. Anatomically, the EACC and MEC rarely extend to opposite sides, and are good candidates for IAIC [18]. The use of IAIC in combination with PBT may be associated with improved clinical outcomes in patients with EACC and MEC.

The present study has several limitations. First, the sample size was small owing to the rarity of these cancers; therefore, the statistical analyses are fragile. Second, the selection criteria for the chemotherapy regimens were inconsistent on a case-by-case basis; additionally, one patient did not receive chemotherapy. Thus, the effects of chemotherapy may have acted as a major confounding factor when evaluating treatment outcomes. Third, this was a retrospective case series study conducted at a single institution. We believe that an analysis with a larger number of cases would therefore be desirable.

PBT resulted in good clinical outcomes and acceptable toxicities in patients with EACC and MEC, including in the setting of combined chemotherapy.

## PRIOR PRESENTATION

The contents of this paper were presented in The 82^nd^ annual Meeting of the Japan Radiological Society on 15 April 2023.
